# Prognostic Factors for Recurrence And Survival of Patients with Breast Cancer Using A Multi-state Model

**DOI:** 10.31661/gmj.v13i.3043

**Published:** 2024-09-17

**Authors:** Maryam Rastegar, Zahra Arab Borzu, Ahmad Reza Baghestani, Roqayeh Aliyari, Ali Akhavan, Anahita Saeedi

**Affiliations:** ^1^ Department of Biostatistics, School of Health, Mashhad University of Medical Sciences, Mashhad, Iran; ^2^ Department of Epidemiology & Biostatistics, Zahedan University of Medical Sciences, Zahedan, Iran; ^3^ Physiotherapy Research Center, Faculty of Paramedical Sciences, Shahid Beheshti University of Medical Sciences, Tehran, Iran; ^4^ Ophthalmic Epidemiology Research Center, Shahroud University of Medical Sciences, Shahroud, Iran; ^5^ Isfahan University of Medical Sciences, Isfahan, Iran; ^6^ Department of Biostatistics, School of Public Health and Health Sciences, University of Massachusetts, Amherst, MA, USA

**Keywords:** Multi-state Model, Survival Probability, Prognostic Factors, Breast Cancer

## Abstract

Background: In many medical studies, patients may experience various events. The analysis in such studies is often administrated using multi-state models. The current study aimed to investigate the effect of risk factors and transition probability on recurrence and death in patients with breast cancer. Materials and Methods: This study was a retrospective cohort study on 814 women with breast cancer admitted to Shahid Ramezanzadeh Radiotherapy Center in Yazd province in Iran between the years 2004 -2012 and were followed until 2016. A multi-state model is applied for data analysis in the R 3.4.1 programming language. Results: Of the 814 patients, 40(5%) experienced recovery after initial treatment and 177(20.7%) experienced the death after initial treatment. For the first year, the transition probabilities from the initial treatment to recovery were estimated at 1.4%, to death was 17% and for recovery to death, it was 29%. The mean sojourn times were estimated as 2.93 and 9.8 years for the treatment and recovery, respectively. Conclusion: Multi-state models predict the transition probabilities in different states of disease, in addition, transition probabilities, mean sojourn time, and hazard ratio in each state can help physicians find suitable care for patients with breast cancer.

## Introduction

Breast cancer is the most common cancer and the second cause leading of mortality among women [1]. According to the worldwide cancer statistics, breast cancer incidence is escalating [2]. More than 8 million individuals are diagnosed with cancer, such that one million of them being breast cancer [3][4]. The incidence of breast cancer was 231000 new cases in the United states in 2015, and 40000 of them experienced death due to breast cancer [5]. Iranian women have a higher prevalence of breast cancer than women in developed countries. About 16% of all cancers in Iranian women are associated with breast cancer, such as it is predicted to be the second cause of mortality in Iran by 2025 [6][7]. Cancer relapse develop in approximately 50% of women with breast cancer at different times after diagnosis of the primary tumor [8].

Breast cancer recurrence of breast cancer is a significant cause of mortality in patients, and overall survival decreases after the occurrence of metastasis [9]. In recent years, many studies have been conducted on the effect of important genetic background, age, and hormonal factors such as large tumor size, lack of estrogen-receptor(ER), Progesterone Receptor(PR), expression, overexpression of human epidermal growth factor receptor (HER2) on recurrence and death due breast cancer [10]. In most non-communicable diseases, there may exist more than one endpoint, for instance, disease-free survival, local recurrence, distant metastasis, or death (which can be defined as an endpoint). In such cases, a separate analysis is used for each of the endpoints. These separate analyses are not appropriate since they do not consider the relationship between different types of events. Recently, methods have been developed that simultaneously model several competing causes of surgery failure or therapy (competing risks models) or that model the development of a patient’s state over time (multi-state models). In multi-state models, several states are defined and the main focus is on the process of transition from one state to another. These models permit the entry of risk factors to make comparisons between factors practicable. Furthermore, it is possible to estimate and compare the impact of risk factors on each stage of the transition [11]. This study aimed to determine the effect of risk factors and transition probability on recurrence and death in Iranian women with breast cancer using the multi-state model.

## Materials and Methods

**Figure-1:**
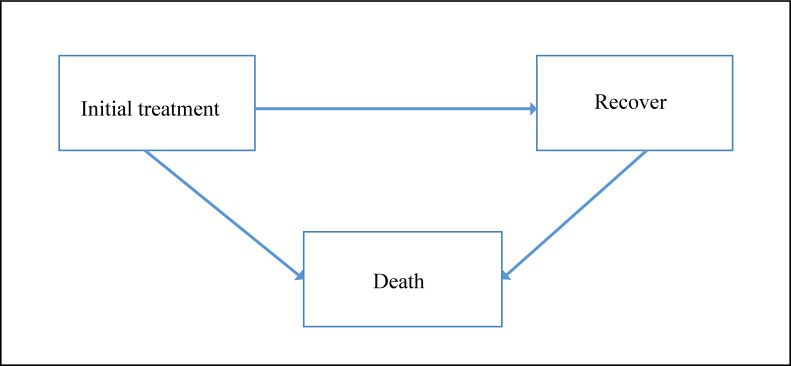
Figure 1. Breast cancer disease transition paths estimated using multi-state model

**Table:** Table1. Baseline characteristics of breast cancer patients.

variable	N (%)
Stage	
Stage I	90(11.1%)
Stage II	404(49.6%)
Stage III+(III&IV)	320(39.3%)
HER2	
Negative	627(77%)
Positive	187(23%)
ER	
Negative	234(28.7%)
Positive	580(71.3%)
PR	
Negative	275(33.8%)
Positive	539(66.2%)
Type surgery	
BCT	260(31.9%)
MRM	554(68.1%)
Lymph node status	
Negative	252(31%)
Positive	562(69%)
Tumor size	
T1	131(16%)
T2	529(65%)
T3+(T3&T4)	154(19%)
	

This is a retrospective cohort study in which 814 women with breast cancer received their first surgery in Shahid Ramezanzadeh Radiotherapy Center, Yazd, Iran between 2004 -2012 and were followed until 2016. The data were obtained from the patient’s medical records by a predetermined checklist including age, and tumor size which was divided into 3 groups: T1 (size<2), T2 (2≤size<5), and T3 (size≥5). Stage in breast cancer is: Abnormal cells are present but have not spread to nearby tissue. Early stage: cancer has spread to other tissue in a small area. Localized: tumor is between 20-50 mm and some lymph nodes are involved or a tumor larger than 50 mm and some lymph nodes are involved or a tumor larger than 50 mm with no lymph nodes involved. Regional spread: the tumor is larger than 50 mm with more lymph nodes involved across a wider region in some cases. There is no tumor present at al. cancer may have spread to the skin or chest wall. Distant spread: cancer has spread beyond the breast to other parts of the body. ER (negative or positive), PR (negative or positive); type of surgery (MRM, BCS); number of metastatic lymph nodes (positive or negative); HER2 (negative or positive); and Antigen ki-67 index as independent variables. The male patients with breast cancer and patients who had experienced just one condition and whose information was not available were excluded from the study. Also, to determine the survival time (whether the patients were dead or alive) of the patients, a phone interview was performed with the permission of both the patients and the hospital.

Statistical Analysis

In the current study, a multi-state model was employed as the main statistical method. As shown in Figure-1, participants could experience the following transitions between states: From initial treatment/surgery (state1) to recovery (state2) or death (state 3) and from recovery to death. Therefore each patient would experience at least one of the states with a transitional probability after receiving initial treatment. We assumed Markov’s continuous-time to estimate the effect of the study covariates on transitions between states. To assess the Markov property, we used the MSM package in R programming language version 3.4.1.

## Results

**Table:** Table2. Transition matrix of breast cancer patients based on the states of disease

condition	Initial Treatment	Recovery	Death
Initial Treatment	597(70%)	40(4.7%)	177(20.7%)
Recovery	0	16(1.8 %)	24(2.8%)
Death	0	0	0

**Table:** Table3. The transition probability matrix in baseline (model without covariate) model for breast cancer data

From	To	T=1st year	T=2nd year	T=3rd year	T=5th year	T=10th year	T=15 th year
Initial Treatment	Initial Treatment	0.96(0.95-0.971)	0.93(0.92-0.94)	0.90(0.88-0.92)	0.85(0.81-0.87)	0.72(0.66-0.76)	0.61(0.56-0.66)
Initial treatment	Recovery	0.014(0.009-0.023)	0.024(0.35-0.64)	0.031(0.02-0.045)	0.038(0.026-0.053)	0.039(0.026-0.057)	0.034(0.02-0.05)
Initial Treatment	Death	0.017(0.012-0.025)	0.037(0.029-0.052)	0.06(0.049-0.082)	0.011(0.09-0.14)	0.23(0.119-0.29)	0.34(0.3-0.41)
Recovery	Recovery	0.71(0.59-0.8)	0.5(0.35-0.64)	0.35(0.2-0.51)	0.181(0.07-0.33)	0.32(0.004-0.1)	0.005(0.0004-0.03)
Recovery	Death	0.29(0.19-0.4)	0.49(0.351-0.648)	0.64(0.48-0.79)	0.81(0.66-0.92)	0.96(0.89-0.99)	0.92(0.90-0.999)

**Table:** Table4. The estimated mean sojourn time (in years) using the multi-state model

Condition	Estimate	Standard Error(SE)	95% CI
Initial treatment	9.8	3.01	(5.25-10.3)
Recovery	2.93	0.66	(1.87-4.5)

Overall, 814 females with BC were studied with an average age of 48.41±12.14 years and the median age was 48 years. The median (Q1-Q3) follow-up time was 5.81 (4.24-8.28) years. The number of patients with one and two occurrences of breast cancer (recovery) were 774(95%) and 40(5%), respectively.

The mean and standard deviation of Ki-67 patients was equal to 9.69±17.82. Most patients were at stage II (49.6%) cancer at the diagnostic time. The percentages of ER+, PR+, Lymph node status+ and HER2- in these patients were 71.3%,66.25,69% and 44.5%, respectively. Sixty-eight percent of patients had type surgery of MRM.

The clinical baseline and demographic characteristics of the patients are listed in Table-1.

In this study, 177 deaths occurred in the initial treatment, and 24 deaths occurred in recovery status Table-2.

The probability of remaining in the previous state in no-recovery patients after 1, 2, 3, 5, 10, and 15 years was 0.96%, 93%,90%,85%,72% , and 61% respectively. Considering a period of 15 years, a patient who is in the initial treatment state would recover with a probability of 3.4% and would die with a probability of 34%. As shown in Table-3. The average sojourn time of patients in each state was obtained from breast cancer patients, so that, it shows the stability of the patients in each stage. The maximum sojourn times considering independent variables related to recovery and death were 2.9 and 7.8 years respectively Table-4.

**Figure-2:**
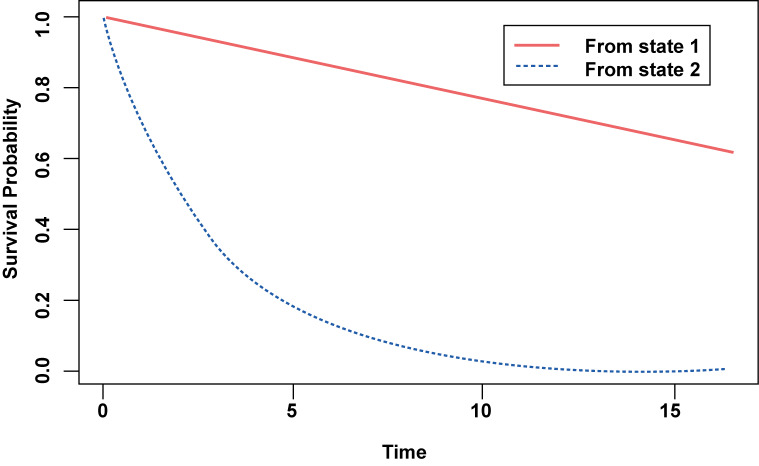
Figure 2. Survival plot for different states of patients with breast cancer

Figure-2. illustrates the prediction of the probability of survival in different states. So, the 10-year survival probability of women with breast cancer in initial treatment was 0.81. Conversely, with recurrence of breast cancer after initial treatment, the survival probability diminishes very quickly to o.o8 approximately.

Table-5 indicates the results of fitting the multi-state model. With an increase in age, the risk of death for women who were in the initial treatment and recovery increased by 1% and 2.7% respectively.

Additionally, with increasing tumor size, the hazard of transition from initial treatment to recovery and death increased to 1.34, and 2.5. Furthermore, the hazard of transition from recovery to death was increased by 94%.

The hazard of transition from initial treatment to recovery for patients with HER2 was 1.38 times compared to patients without HER2. The hazard of transition from recovery state to death for patients with HER2 was 11% less than patients without HER2. The hazard of transition from initial treatment and recovery to death for patients with Breast-conserving therapy (BCT) was 55%, and 67% less than patients with modified radical mastectomy (MRM). A one-unit increase in Ki-67 decreased the hazard of transition from initial treatment and recovery to death by 2%, and 4% respectively.

The goodness-of-fit baseline model was evaluated by the Pearson test (P-value=0.25).

## Discussion

**Table:** Table5. Hazard ratio (95% CI) of risk factors associated with breast cancer using multi-state. model

	Initial treatment- recovery	Initial treatment- death	Recovery- death
Age of patients	0.99(0.97-1.02)	1.01(1-1.032)	1.027(0.99-1.06)
HER2	1.38(0.76-2.51)	0.79(0.4-1.76)	0.76(0.23-1.37)
PR	1.75(0.69-4.41)	0.49(0.22-1.08)	0.89(0.77-8.75)
ER	0.29(0.11-0.72)	0.67(0.55-0.91)	0.93(0.29-3)
Type of surgery(BCT vs MRM)	1.01(0.42-2.45)	0.45(0.1-1.98)	0.33(0.13-0.83)
Stage	1.38(1.05-1.8)	1.22(0.92-1.61)	1.8(0.57-2.11)
Ki-67	0.99(0.98-1.017)	0.98(0.96-1.03)	0.96(0.94-0.99)
Tumor size	1.34(1.1-2.65)	2.5( 1.05-3.54)	1.94(0.56-2.36)
Number of involved lymph nodes	1.24(0.67-3.24)	1.15(0.78-2.9)	0.98(0.45-1.18)

The present study aimed to investigate a multi-state model and its application to women with breast cancer. Multi-state models are useful tools in the analysis of survival data with more than one endpoint. These models could predict the probability of transition between the stages of a disease and assess the effect of prognosis factors on the various states [11].

Constructing multi-state models sheds light on the associations between the different endpoints, such as recurrence or relapse and survival in breast cancer.

In this study, we considered the effect of the clinical and pathological characteristics of the patients. Furthermore, we obtained the probability of transitions among various states for the patients at 1 to 15 years. As estimated, the probabilities of transitions in the first year, from the initial treatment to recovery and from recovery to death were 1.4% and 29%, respectively. After 15 years, the estimated probabilities of transition from initial treatment to recovery and recovery to death reached 3.4% and 92% respectively.

Sojourn time is considered a measurement that determines mean sojourn times in each transient state for a given set of independent variables, the maximum estimated mean sojourn times for initial treatment and recovery states were 9.8 and 2.93 years, respectively.

In this study, there were three states: initial treatment, recovery, and death as the first, second, and, absorbing states, respectively. About 40 recoveries occurred in the initial treatment state and 24 patients were transferred from the recovery state to the death state and 177 patients, were directly transferred to the death state.

The findings of this study revealed that the increase in age at diagnosis has no significant association with recovery and death, which is in line with the study of Putter et al. And Babaee et al. [11][12]. Baghestani et al. proved a significant relationship between age and recovery [13]. However, the results of a previous study showed that the survival rate decreased with an increase in age [14]. HER2+ and PR+ variables were associated with the increased hazard of recovery such that, PR+ and HER2+ status increased the risk of recovery by 38% and 75%, respectively. While they decreased the hazard of death after initial treatment and recovery. In this regard, different results have been reported in the previous studies, some of them are in line and some of them are not consistent with our findings [2][13][14][15][16][17]. In our study, ER+ increased the risk of death after initial treatment by about 31% and 7% after initial treatment and recovery and decreased the risk of recovery by 71% after initial treatment. In the study by Farahani et al. ER+ increased the recovery after initial treatment and decreased mortality [2]. We found that the type of surgery BCT increased recovery by 1% and decreased death after initial treatment and recovery by 55% and 67%, respectively. This finding is in line with the results of previous studies [13].

In this study, with the increasing stage and tumor size of breast cancer, the hazard of transition from initial treatment to recovery and from initial treatment and recovery to death was increased. The results of several previous studies confirmed our findings [2][13][18][19][20].

We discovered that increasing Ki-67 decreased the hazard of transitions from initial treatment to recovery and from initial treatment and recovery to death. The study of Kanyilmaz et al. Showed a significant association between survival and the Ki-67 index [21]. The results of this study indicated that in the presence of lymph nodes, the hazard of death in the transition from initial treatment to recovery and death increased by 24% and 15%, respectively. In contrast, the risk of mortality from recovery to death decreased by 2%. Many studies have shown that an increase in lymph node involvement is associated with an increase in recovery and mortality [13][22][23]. Our study had several limitations. Access at a higher level to the details of a dataset is often required in the multi-state model structure. Applying the multi-state models may be laborious or unfeasible for diseases with multiple simultaneous transition pathways. Additionally, due to low transition numbers, the statistical power of our model may be lower compared to other studies with multiple transitions and similar designs. However, maybe breast cancer was not the main reason for the transition from (recovery to death) or (initial treatment to death) after 5 or 10 years. So, more studies are needed in this field.

## Conclusion

We estimated the probabilities of transition and hazard ratios of transition in each state using a multi-state model. The interpretation of estimated hazard ratios for different estimations in multi-state models may not be clinically unchallenging. Multi-state models presented important information on disease outcomes in different transitions. In this study, we illustrated the transition paths of breast cancer and estimated transition probabilities, mean sojourn time, and hazard ratios in each state. The findings of our study can be utilized to suggest appropriate care for patients with breast cancer.

## Acknowledgement

Thanks to Shahid Ramezanzadeh Radiotherapy Center, Yazd, Iran. This study was approved by the Ethics Committees of the Shahid Beheshti University of Medical Science, Tehran, Iran (no: IR.SBMU.RETECH.REC.1400.854)

## Conflict of Interest

None
